# Assessment of Uncarboxylated Osteocalcin Levels in Type 2 Diabetes Mellitus

**DOI:** 10.7759/cureus.35297

**Published:** 2023-02-22

**Authors:** Taghreed M Alamri, Fahad A Alhumaydhi, Afshan Z Wasti

**Affiliations:** 1 Medical Laboratories, College of Applied Medical Sciences, Qassim University, Qassim, SAU

**Keywords:** type 2 diabetes mellitus, carboxylated osteocalcin, glucose metabolism, osteocalcin, uncarboxylated osteocalcin

## Abstract

Osteocalcin is one of the main organic components of the bone matrix and consists of 49 amino acids excreted from osteoblastic cells in carboxylated and uncarboxylated forms. Carboxylated Osteocalcin belongs to the bone matrix, whereas uncarboxylated osteocalcin (ucOC) is an important enzyme of osteocalcin in the circulatory system. It is an essential protein for balancing the minerals in bones, binding with calcium, and regulating body glucose levels. In this review, we point out the assessment of ucOC levels in type 2 diabetes mellitus. The experimental results that show ucOC controls glucose metabolism are significant because they relate to the current obesity, diabetes, and cardiovascular disease. To confirm that, low serum levels of ucOC were a risk factor for poor glucose metabolism, and further clinical studies are required.

## Introduction and background

An increased blood sugar level is a symptom of a range of metabolic illnesses known as diabetes mellitus (DM) that affect insulin secretion, insulin action, or both. Long-term harm and dysfunction of many organs, particularly the kidneys, heart, nerves, eyes, and blood vessels, are linked to DM. Development of DM involves a variety of disease-causing events, including autoimmune damage of pancreatic beta cells through subsequent insulin action [1] . In Saudi Arabia, DM is one of the major health issues, and it is ranked among the top 10 countries in the world regarding the prevalence of DM [2]. Type 2 DM is characterized by insulin resistance because dysfunction of beta cells. DM patients suffer an impairment in the action or secretion of insulin. The illness develops when the body does not release enough insulin or when the body's cells are unable to use insulin [3].

Osteocalcin (OCN) was the first molecule to be identified as a connection between bone metabolism and glucose [4]. One of the major organic forms of the bone matrix is OCN [5]. In the bones, OCN binds to hydroxyapatite after carboxylation of glutamyl residue at positions 17, 21, and 24 γ- in the presence of vitamin K [6]. Carboxylated osteocalcin (COC) contributes to the outer bone matrix.

In contrast, uncarboxylated osteocalcin (ucOC) is the circulatory system's active form of OCN. It is an essential protein for balancing bone minerals, binding with calcium, and regulating body glucose levels. The uncarboxylated form, which is secreted into the bloodstream, promotes insulin secretion and plays role in glucose balance [7]. Contrarily, insulin increases the expression of OCN in osteoblasts. [8]. When the pH is acidic enough to decarboxylate proteins, bone resorption takes place. Osteoclasts analyze the carboxylation status and work of OCN. Accordingly, bone resorption-dependent glucose metabolism in mice and humans is promoted or inhibited by raising or lowering insulin signaling in osteoblasts [9]. According to recent publications that are primarily based on rat models and according to in vitro research, the noncarboxylated form of OCN modulates physiological pathways in an endocrine manner [10].

## Review

Material and methods

The aim of this study is to investigate the relationship between ucOC levels and type 2 DM. We thoroughly searched the following databases: PubMed, EMBASE, and Cochrane. We examined English-language publications published between 1989 and 2018. Likewise, we looked through the reference lists of the retrieved papers to find more relevant material.

**Figure 1:**
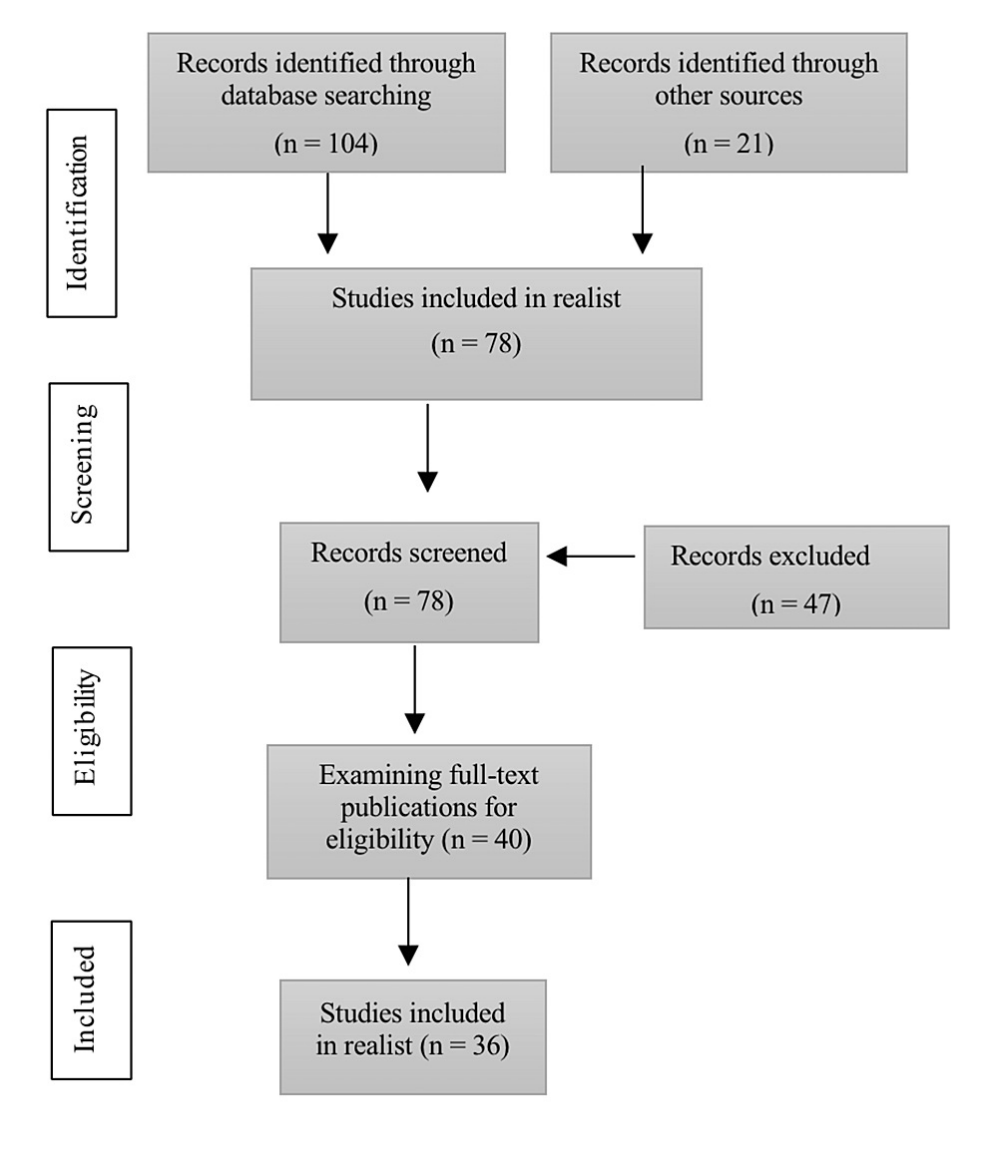
The mechanism for selecting records related to the study according to the PRISMA guidelines PRISMA, Preferred Reporting Items for Systematic Reviews and Meta-Analyses

Keywords included “uncarboxylated osteocalcin,” “osteocalcin,” “carboxylated osteocalcin,” “diabetes mellitus type II,” “glucose metabolism.” The first search yielded 125 papers. However, after the screening and quality assessment process based on abstract and full-text documents, only 35 articles were included (Figure 1). This review was performed according to the PRISMA (Preferred Reporting Items for Systematic Reviews and Meta-Analyses) guidelines [11].

Osteocalcin

In general, the skeleton is responsible for an organism's support and movement. Beyond its mechanical capabilities, bone has come to be recognized as a regulator of a number of metabolic processes separate from mineral metabolism [7]. It is regulated in osteoblast in response to physiological or pathological processes. It can be affected by growth factor, hormone, cytokines, and physical stimulus through signal transduction pathway, bonding to the *BGLAP* gene promoter or interfacing with nuclear transcription factors. A recent study reported that ucOC enhances mice's insulin sensitivity and glucose tolerance, minimizing the onset of DM. Additionally, ucOC raised the expression and production of adiponectin in adipose tissue, which improved insulin sensitivity. Moreover, decreased circulating levels of adiponectin have been endemically linked to insulin resistance and type 2 DM. Adipocytes' expression of adiponectin is raised by GluOC. However, results in human studies are conflicting and inconsistent; as compared to healthy adults, people with type 2 DM had significantly lower serum levels of OCN. Also, serum OCN in some investigations is inversely correlated with the homeostasis pattern of insulin resistance index, fasting plasma glucose, and fasting insulin [12]. Numerous drugs that are utilized in both clinical and non-clinical contexts have an impact on OCN levels and may be useful in the treatment of type 2 DM. Thorough research should be conducted into the molecular processes that control OCN expression and its potential significance in the development and management of DM [13].

Uncarboxylated osteocalcin

The active form of OCN, known as ucOC, is released by osteoblasts and circulates in the blood. Reverse COC remains in the bones [14]. Several studies agreed that the protein receptor GPRC6A is a putative receptor for ucOC [15-17] and is known for its wide distribution in many tissues of the human body as the pancreas. Many studies have reported its association with insulin secretion and insulin resistance, which leads to the development of type 2 DM [18-23].

Osteocalcin and insulin receptors on osteoblasts

**Figure 2:**
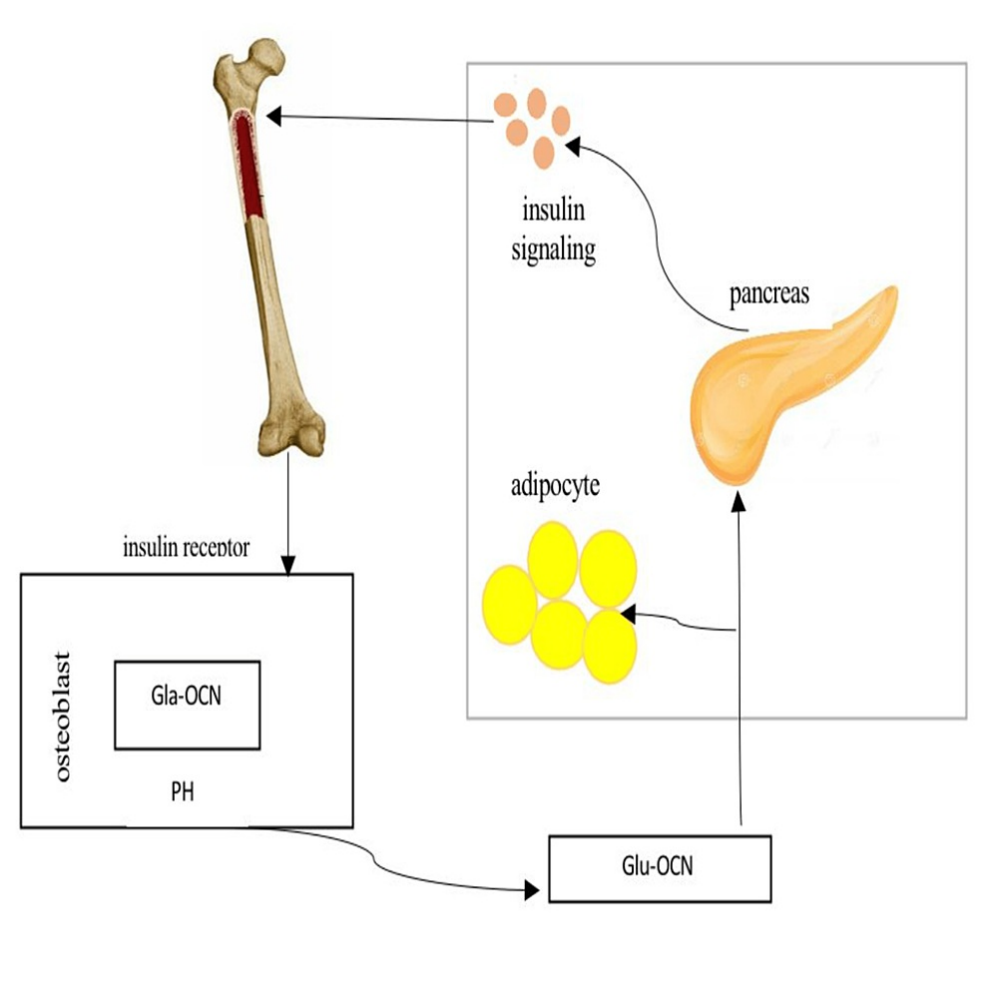
Osteocalcin and insulin receptors on osteoblasts Gla-OCN, carboxylated osteocalcin; Glu-OCN, uncarboxylated osteocalcin

Lately, separate reports from the Karsenty Lab [24,9] and the research group of Clemens [25,26] indicate that osteoblasts regulate glucose metabolism through insulin signaling via the ucOC pathway (Figure 2). Rats with osteoblast-specific insulin receptor deletion (InsRosb/) had high blood sugar, decreased insulin secretion, and low ucOC levels [24]. In addition, the experimental results from that study showed that OCN was decarboxylated in resorption lacunae, which increased the levels of circulating ucOC, and that insulin signaling in osteoblasts increased bone resorption by osteoclasts [24]. These results are backed up by a separate report showing that perturbation of insulin receptors in osteoblasts decreases total OCN and ucOC levels, leading to glucose intolerance and reduce insulin levels in rats [25]. Therefore, in these rodent models, insulin acts through insulin receptors present in osteoblasts and thus increases the decarboxylation of OCN. These results illustrate new mechanisms that participate in insulin signaling and regulation of glucose metabolism and focus attention on the need to consider bone in turnover with ucOC [27]. The detection of more variables influencing this pathway is anticipated, and further explanation is needed given the importance of this mechanism for human skeletal and glucose metabolism [28,29].

The results obtained that show ucOC regulates glucose metabolism are significant because they relate to DM and heart disease. Obesity is a risk factor for DM and cardiovascular disease, as it leads to insulin resistance [30]. Therefore, new strategies to improve insulin resistance may help lower the prevalence of DM [31].

Scientific evidence from knockout rats and cells points out that ucOC promotes insulin secretion and improves insulin sensitivity [8,31]. Reduced total OCN levels are linked to insulin resistance, elevated blood sugar, and type 2 DM in humans, according to observational studies. [32]. In a recent study, decrease total OCN (TOC) levels were associated with high prevalence of metabolic syndrome, elevated blood glucose, and triglyceride levels, and remained significant after glucose control [33]. Nevertheless, research wherein ucOC and TOC were assayed is smaller in number and provided the most effective certified proof of a function for ucOC specifically. Therefore, extensive observational research is needed to evaluate ucOC [34]. Cross-sectional studies, which only indicate associations at a single point in time, make it challenging to determine causality. Consequently, longitudinal research determining the role of TOC and ucOC as independent predictors of onset DM and cardiovascular problems would be very important. When the role of ucOC in human metabolism has been more precisely characterized, more research will be required to determine how to modify ucOC levels to affect clinical outcomes [35]. The function of osteoclasts contributes to bone resorption and provides an acidic resorption gap where OCN is decarboxylated. An exciting attitude at the interplay in fracture hazard and DM [36].

## Conclusions

We conclude that low serum ucOC is a risk factor for glucose metabolism and the development of type 2 DM. We need more studies on humans. If these effects are proven, they will help in the prevention and treatment of DM.
